# The Association Between Dexmedetomidine and Bradycardia: An Analysis of FDA Adverse Event Reporting System (FAERS) Data and Transcriptomic Profiles

**DOI:** 10.3390/genes16060615

**Published:** 2025-05-22

**Authors:** Robert Morris, Suguna Aishwarya Kuppa, Xinran Zhu, Kun Bu, Weiru Han, Feng Cheng

**Affiliations:** 1 Department of Pharmaceutical Science, Taneja College of Pharmacy, University of South Florida, Tampa, FL 33613, USA; rpm4@usf.edu (R.M.); skuppa@usf.edu (S.A.K.); xinranzhu@usf.edu (X.Z.); 2 Department of Mathematics & Statistics, College of Art and Science, University of South Florida, Tampa, FL 33620, USA; kunbu@usf.edu (K.B.); weiruhan@usf.edu (W.H.)

**Keywords:** FAERS, pharmacovigilance, drug interactions, bradycardia, RNA-seq, dexmedetomidine, association rules, DDI

## Abstract

Background/Objectives: Bradycardia, an uncharacteristically low heart rate below 60 bpm, is a commonly reported adverse drug event (ADE) in individuals administered dexmedetomidine for sedation. Dexmedetomidine is frequently used as a sedative and analgesic for both intubated and non-intubated patients due to its low risk of respiratory depression. The purpose of this study was to further characterize the safety profile of dexmedetomidine using safety reports collected from the FDA Adverse Event Reporting System (FAERS) and transcriptomic data. Methods: Association rule mining was used to both identify additional ADEs that presented concurrently with bradycardia in patients sedated with dexmedetomidine, as well as to characterize potential drug–drug interactions (DDIs). Furthermore, public transcriptomic data were analyzed to identify differentially expressed genes that may elucidate the genetic drivers of elevated bradycardia risk in those administered dexmedetomidine. Results: Bradycardia was the most frequently reported ADE for individuals administered dexmedetomidine. Other cardiovascular-related ADEs commonly associated with bradycardia included syncope (lift = 4.711), loss of consciousness (lift = 3.997), cardiac arrest (lift = 2.850), and hypotension (lift = 2.770). Several possible DDIs were identified, including Lactated Ringer’s solution (lift = 5.441), bupivacaine (lift = 2.984), and risperidone (lift = 2.434), which may elevate bradycardia risk. Finally, eight genes related to cardiac muscle contraction were identified as possible regulators of dexmedetomidine-induced bradycardia, including *COX5B*, *COX6A2*, *COX8B*, *MYH7*, *MYH6*, *MYL2*, *UQCRQ*, and *UQCR11* in mouse cardiac cells. Conclusions: Key clinical takeaways include the co-presentation of multiple cardiovascular ADEs, including cardiac arrest, hypotension, and syncope, alongside dexmedetomidine-associated bradycardia. Furthermore, several possible DDIs with dexmedetomidine were also identified.

## 1. Introduction

Bradycardia is a commonly occurring cardiac arrhythmia characterized by a prolonged and uncharacteristically low heart rate below 60 beats per minute (bpm) that is typically diagnosed through visualization of an abnormal electrocardiogram [1,2,3]. Bradycardia frequently arises due to complications in pulse conduction by the sinoatrial node, the primary pacemaker and regulator of heart rate, although other factors including myocardial infarction, congenital heart defects, certain medications, myocarditis, and blockages of atrioventricular conduction may also contribute [1,2,4]. Although many cases of bradycardia may present asymptomatically, particularly in those with high fitness levels or those during periods of rest, unstable bradycardia may result in significant fatigue, syncope, dizziness, confusion, chest pain, and cardiac arrest [1,2,5]. For patients presenting with sinus bradycardia and hemodynamic instability, intravenous treatment of 0.5 mg atropine for up to 30 min is the standard treatment modality; however, for patients that do not respond to atropine therapy and continue to present with heart rate abnormalities, a temporary pacemaker may be considered [1,2,6].

Dexmedetomidine, first approved for clinical use by the Food and Drug Administration (FDA) in 1999, is a highly selective α2-adrenergic receptor agonist that is commonly used as a sedative in clinical settings [7,8]. It suppresses sympathetic nervous system activity and reduces the release of norepinephrine, resulting in decreased blood pressure and the onset of sleep [8,9]. Dexmedetomidine is indicated for use in the sedation of intubated and mechanically ventilated patients, as well as for peri-operative use in non-intubated patients [8,10,11]. For intubated patients, the typical dosage ranges from 0.2 to 0.7 mcg/kg per hour; however, dosages upwards of 1.5 mcg/kg per hour are considered safe and are associated with minimal increases in side effects [8]. For anesthetic purposes in non-intubated patients, a loading dose of 0.5–1.0 mcg/kg is administered first followed by an hourly administration of 0.2–0.7 mcg/kg [8,12,13]. Regardless of patient status, manufacturers advise that dexmedetomidine administration does not exceed a 24 h period [8].

Because of its 1600:1 selectivity for α2-adrenergic receptors compared to the α1 isoform, dexmedetomidine is associated with reduced deleterious effects on respiratory function [8,13]. In addition, pretreatment with dexmedetomidine has demonstrated cardioprotective effects against ischemia–reperfusion injury (IRI) by stabilizing cardiac electrophysiology and modulating inflammatory signaling pathways [8,14,15]. Despite this, multiple studies have demonstrated a significant correlation between dexmedetomidine treatment and the risk of bradycardia [16,17,18,19,20]. A meta-analysis and systematic review of 15 studies indicating performance of laparoscopic surgical procedures found that individuals treated with dexmedetomidine during surgery had an approximately three-fold increase in relative risk (RR = 2.81, 95% CI 1.34–5.91) of developing intraoperative bradycardia compared to those not sedated with dexmedetomidine [20]. Another meta-analysis of 18 studies collected between 2003 and 2016 found a nearly 26-fold increase in relative risk (RR = 26.3, 95% CI 16.3–42.4) of developing bradycardia in those sedated with dexmedetomidine compared to untreated controls [15]. Finally, a disproportionality analysis found that dexmedetomidine was associated with a near 57-fold increase (ROR = 56.66, 95% CI 49.90–64.35) in the likelihood of bradycardia [21]. Thus, despite its efficacy and reduced association with impaired respiratory function, it is important to explore the safety profile of dexmedetomidine and characterize the associated bradycardia risk, particularly for those with pre-existing cardiovascular complications and arrhythmias.

To address these gaps in dexmedetomidine characterization, this study employed a multidisciplinary approach combining the disproportionality analysis of safety reports derived from the FDA Adverse Event Reporting System (FAERS) with transcriptomic analysis of a public RNA-seq dataset to further elucidate the correlation between dexmedetomidine treatment and bradycardia risk. FAERS is one of the largest post-marketing drug surveillance databases available globally with over 30 million adverse event reports currently collected and an additional 1 million reports added annually [3,22,23]. FAERS provides an example of real-world data that more effectively captures the heterogeneity of patient experience while RNA-seq data provide experimental data that supplement the findings derived from FAERS analysis. When coupled together, a more robust conclusion can be drawn as to the association between dexmedetomidine and bradycardia if their directionality is comparable. Furthermore, association rule mining was used to identify concurrent adverse events that most frequently manifested alongside bradycardia in those treated with dexmedetomidine as well as to identify possible drug–drug interactions (DDIs). Finally, the potential underlying genetic mechanisms driving this elevated bradycardia risk were also explored.

## 2. Materials and Methods

### 2.1. FAERS Data

Adverse event reports submitted through FAERS were accessed using the publicly available FAERS dashboard tool. Each record derived from FAERS contains the following seven distinct data entries:

(1) Drug information;
(2) Drug-related adverse events (ADEs);
(3) Patient outcomes for each indicated ADE;
(4) Demographic information, including patients’ sex and age;
(5) Origin of the reported ADE;
(6) Date of submission;
(7) Indications for use of each indicated drug.

Reports were extracted from the FAERS dashboard using both the generic drug term (dexmedetomidine and dexmedetomidine hydrochloride) and the most common brand name (Precedex) as input search terms. A report in which either the generic or brand name of the drug of interest was indicated in either the ‘suspect product names’ or ‘suspect product active ingredients’ data columns was eligible for inclusion in this study.

### 2.2. Removal of Duplicate FAERS Reports

The majority of the over 30 million records accessible through the FAERS dashboard are direct reports, indicating that the FDA was notified of the occurrence of an ADE through a submission made by a patient, healthcare provider, pharmaceutical company, or drug manufacturer. However, a relatively small subset of these reports may instead be indirect in nature. That is, rather than being directly submitted through the FAERS portal by an individual, some reports may be extracted from publications in the literature. As a result, multiple reports indicating the same ADE for the same patient albeit with different case IDs may arise in the database as multiple sources may identify and report the event after a review of the literature. To avoid the adverse effects of including spurious reports in the final analysis, any indirect reports extracted from publications were ultimately excluded from analysis for two reasons. First, some reports may be duplicated more than 10 times for the same patient-adverse event pair. By excluding these indirect reports, the strength of the potential safety signal is stabilized, and the elevated risk of false positive generation induced by inflated case counts is attenuated. Next, inconsistencies may be present among reports, either as a result of typographical errors, differences in interpretations, differing study goals, or direct exclusion of potentially relevant information. Data analysis was performed using R statistical software version 4.3.2.

### 2.3. Association Rule Mining

Next, association rule mining was used to identify additional co-presenting ADEs that were commonly reported alongside dexmedetomidine-associated bradycardia. Each association rule was analyzed using the arules package in R statistical software. The following formulas were used to calculate the support, confidence, and lift values for each association rule:

(1) $$\mathrm{Support}\left(\left\{X\right\}\to \left\{Y\right\}\right)=\frac{\mathrm{Number}\;\mathrm{of}\;\mathrm{reports}\;\mathrm{containing}\;X\;\mathrm{and}\;Y}{\mathrm{Total}\;\mathrm{number}\;\mathrm{of}\;\mathrm{reports}}$$

(2) $$\mathrm{Lift}\left(\left\{X\right\}\to \left\{Y\right\}\right)=\frac{\mathrm{Support}\left(X\to Y\right)}{\mathrm{Support}\left(X\right)\times \mathrm{Support}\left(Y\right)}$$

(3) $$\mathrm{Confidence}\left(\left\{X\right\}\to \left\{Y\right\}\right)=\frac{\mathrm{Number}\;\mathrm{of}\;\mathrm{reports}\;\mathrm{containing}\;X\;\mathrm{and}\;Y}{\mathrm{Number}\;\mathrm{of}\;\mathrm{reports}\;\mathrm{containing}\;X}$$

Despite its popularity in identifying significant patterns among data, use of the lift metric in association rule mining presents several limitations. First, the use of an arbitrary cutoff value to determine the significance of a given rule may lead to erroneous interpretations, particularly in datasets with skewed distributions or for those with low confidence (rare itemsets). Furthermore, the standard lift value does not provide confidence intervals or *p*-values, thus making it difficult to assess the statistical validity of observed associations deemed significant based on the cutoff criteria. Consequently, rules with high lift values may arise simply as a result of the sampling variability and not due to a true significant association among items. Thus, a complementary validation method such as disproportionality analysis is needed to differentiate significant rules from spurious rules arising by chance.

Disproportionality analyses evaluate the statistical significance of associations by comparing their observed frequencies against the expected frequency under the assumption of statistical independence. By applying this analysis, the robustness and statistical relevance of each association rule can be ascertained and the likelihood of false positive signals can be reduced.

The disproportionality analysis calculates the odds ratio and corresponding confidence intervals (CIs) using a standard 2 *×* 2 contingency table. For the identification of bradycardia-associated ADEs, referred to as ADE 2, the following table is used (Table 1):

For the identification of significant DDIs with dexmedetomidine-associated bradycardia, the following table is used (Table 2):

The odds ratio and corresponding CI for each rule were calculated using the fisher.test function in R.

### 2.4. Transcriptomic Data Analysis

Finally, a public transcriptomic dataset from the NCBI GEO database (GEO id: GSE210873) was analyzed to explore the genes in mouse-derived apical cardiac cells regulated by the administration of dexmedetomidine [24]. In this dataset, mouse-derived cardiac tissue was treated intravenously with either a preconditioning Yohimbine vehicle and 10 μg/kg of dexmedetomidine (treatment group) or just the preconditioning vehicle control group. Briefly, mice cardiac tissue was subjected to myocardial ischemia for 30 min followed by reperfusion for 120 min. In the treatment group, the preconditioning vehicle was administered 30 min prior to dexmedetomidine administration, and all drugs were intravenously delivered through the caudal vein for 10 min. Three replicates were available for both the control and treatment groups. Raw sequencing files in FASTQ were extracted to the online Galaxy interface and then aligned to the mm10 mouse genome using the HISAT2 alignment tool. Count tables were subsequently generated using the featureCounts tool V2.2.1, and differentially expressed genes were identified using DESeq2. This list of differentially expressed genes was then used as an input to identify enriched disease pathways using the ShinyGo V0.81 web tool.

## 3. Results

### 3.1. Top 10 ADEs of Dexmedetomidine Treatment

First, the top 10 most frequently reported ADEs were identified for patients administered the sedative medication dexmedetomidine. After removal of indirect reports, a total of 1611 reports were included in the analysis. As shown in Table 3, the most frequently reported ADE among these 1611 cases was bradycardia (n = 199) followed by hypotension (n = 124), cardiac arrest (n = 118), off-label use (n = 105), agitation (n = 67), drug ineffective (n = 58), drug interactions (n = 56), product use issues (n = 53), oxygen saturation decreased (n = 52), and pyrexia (n = 48).

### 3.2. ADEs Associated with Bradycardia

Next, association rule mining was used to identify other dexmedetomidine-associated ADEs that frequently co-occurred alongside bradycardia in patients administered the sedative. A lift value greater than one indicates that bradycardia and a given ADE were indicated together more frequently than the expected number of instances. A significant bradycardia–ADE pair was defined as one with a lift cutoff ≥1.5 and a minimum count of 10. As shown in Table 4, the most significant ADEs that commonly co-presented alongside bradycardia were syncope (lift = 4.711), loss of consciousness (lift = 3.997), cardiac arrest (lift = 2.850), hypotension (lift = 2.770), overdose (lift = 2.336), drug interaction (lift = 2.148), product administered to patient of inappropriate age (lift = 1.804), cardio-respiratory arrest (lift = 1.793), and respiratory arrest (lift = 1.562).

To validate the potential associations identified through association rule mining, a disproportionality analysis was also conducted. As shown in Table 5, syncope (OR = 11.04, 95% CI: 3.86–33.97), loss of consciousness (OR = 7.65, 95% CI: 3.28–17.98), cardiac arrest (OR = 4.71, 95% CI: 3.06–7.19), hypotension (OR = 4.54, 95% CI: 2.98–6.85), overdose (OR = 3.01, 95% CI: 1.26–6.69), and drug interactions (OR = 2.73, 95% CI: 1.43–4.99) were identified as significant potential co-occurring ADEs. Conversely, product administered to a patient of inappropriate age (OR = 2.11, 95% CI: 0.95–4.32), cardio-respiratory arrest (OR = 2.08, 95% CI: 0.90–4.41), and respiratory arrest (OR = 1.76, 95% CI: 0.91–3.22) were not considered statistically significant association rules, as their odds ratio confidence intervals included one.

### 3.3. Possible DDIs with Dexmedetomidine

In addition to identifying common ADEs that co-occurred with bradycardia, association rule mining was also used to identify potential DDIs. Drugs co-administered with dexmedetomidine that were associated with bradycardia more frequently than expected were selected as candidate interactions. A significant DDI was defined as an association rule including dexmedetomidine–drug pair and bradycardia with a lift cutoff ≥ 1.5 and a minimum count of 10. As shown in Table 6, the top DDIs associated with bradycardia were Lactated Ringer’s solution (lift = 5.441), bupivacaine (lift = 2.984), risperidone (lift = 2.434), albuterol (lift = 1.927), potassium chloride (lift = 1.587), haloperidol (lift = 1.579), and sevoflurane (lift = 1.513).

To validate these potential associations identified through association rule mining, a disproportionality analysis was also conducted. As shown in Table 7, Lactated Ringer’s solution (OR = 16.96, 95% CI: 5.49–62.19), bupivacaine (OR = 4.43, 95% CI: 1.93–9.78), risperidone (OR = 3.22, 95% CI: 1.46–6.74), and albuterol (OR = 2.32, 95% CI: 1.11–4.53) were identified as significant potential DDIs. Conversely, potassium chloride (OR = 1.79, 95% CI: 0.90–3.35), haloperidol (OR = 1.79, 95% CI: 0.96–3.17), and sevoflurane (OR = 1.71, 95% CI: 0.98–2.86) were determined to not be significant association rules.

### 3.4. Genes Regulated by Dexmedetomidine in Mouse Cardiac Cells

**Figure 1:**
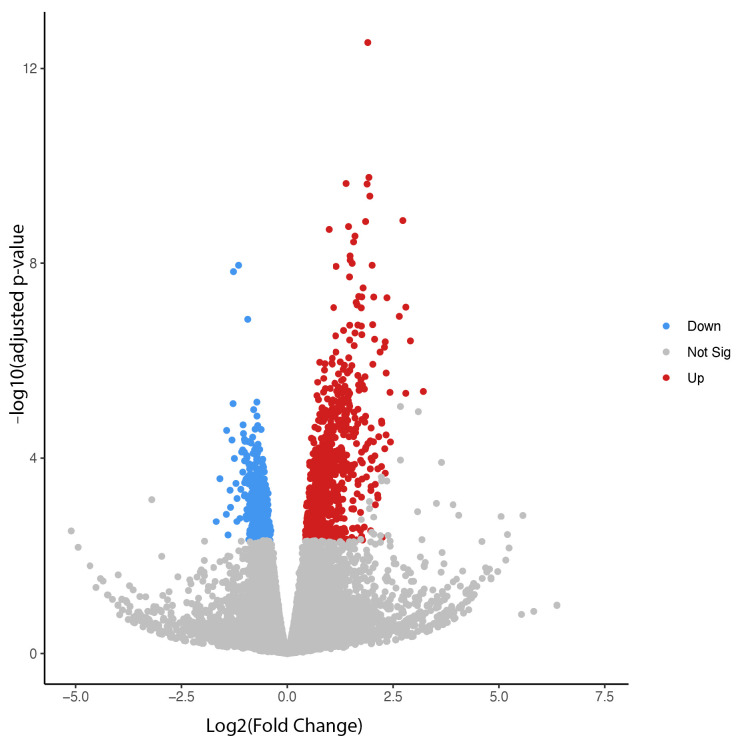
Volcano plot for differentially expressed genes between dexmedetomidine treatment and control at *p* ≤ 0.05.

Transcriptomic analysis was conducted using the publicly available RNA-seq dataset (GEO ID: GSE210873) to identify key regulatory genes differentially expressed between treatment and control groups that may contribute to drug-induced bradycardia. In this dataset, primary mouse-derived cardiac tissue was intravenously treated with either a preconditioning Yohimbine vehicle alone (control group) or the same vehicle followed by 10 μg/kg of dexmedetomidine (treatment group). As shown in Figure 1, a total of 950 genes were differentially expressed between the two groups, including 702 upregulated and 248 downregulated genes, based on an adjusted *p*-value < 0.05 and a fold change > 1.5.

### 3.5. Disease Pathways Enriched in Dexmedetomidine-Treated Cardiac Cells

**Figure 2:**
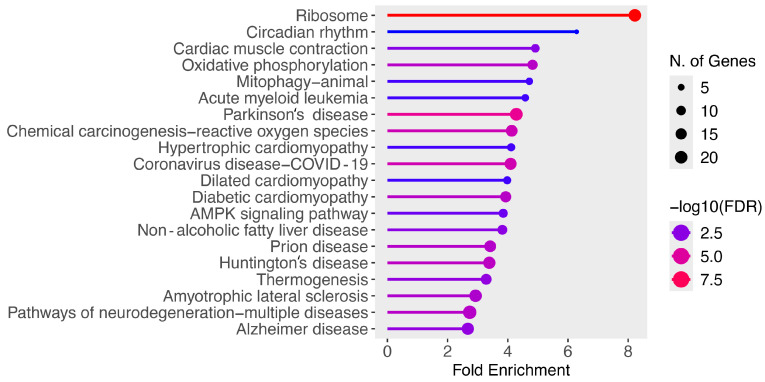
Enriched pathways for downregulated genes at FDR ≤ 0.05.

Finally, the list of differentially expressed genes was used as an input for the ShinyGO V0.81 enrichment tool in order to identify disease and molecular pathways that were enriched in dexmedetomidine-treated cardiac cells relative to the control. Figure 2 presents all of the enriched pathways for downregulated genes in dexmedetomidine-treated mouse cardiac cells with a false discovery rate (FDR) less than or equal to 0.05. As shown in Table 8, the top 10 enriched pathways for downregulated genes were ribosome (n = 20 genes; fold enrichment (FE) = 8.22), circadian rhythm (n = 4 genes; FE = 6.29), cardiac muscle contraction (n = 8 genes; FE = 4.92), oxidative phosphorylation (n = 12 genes; FE = 4.82), mitophagy-animal (n = 6 genes; FE = 4.72), acute myeloid leukemia (n = 6 genes; FE = 4.58), Parkinson’s disease (n = 21 genes; FE = 4.28), chemical carcinogenesis-reactive oxygen species (n = 17 genes; FE = 4.13), hypertrophic cardiomyopathy (n = 7 genes; FE = 4.11), and coronavirus disease COVID-19 (n = 18 genes; FE = 4.09). With regard to bradycardia, eight downregulated genes were mapped to the cardiac muscle contraction pathway including *COX5B*, *COX6A2*, *COX8B*, *MYH7*, *MYH6*, *MYL2*, *UQCRQ*, and *UQCR11.*

## 4. Discussion

A multidisciplinary approach was utilized in this study to assess the risk of bradycardia in patients treated with dexmedetomidine, as well as to further characterize the safety profile of dexmedetomidine by using association rule mining to identify potential co-presenting ADEs and DDIs. Furthermore, RNA-seq analysis was performed to identify possible genetic drivers of the observed elevated risk of bradycardia in those administered the sedative dexmedetomidine. Bradycardia was found to be the most commonly reported ADE for patients sedated with dexmedetomidine. Other cardiovascular-related ADEs that commonly co-presented with bradycardia in those sedated with dexmedetomidine included syncope (lift = 4.711), loss of consciousness (lift = 3.997), cardiac arrest (lift = 2.850), and hypotension (lift = 2.770). Ideally, anesthesia minimizes patient pain and discomfort by inhibiting brain function while maintaining cardiac activity [25,26]. Clinically, suppression of cardiac function in patients undergoing surgery may lead to complications, thus considerations for pre-existing conditions and patient cardiovascular health should be made prior to the use of dexmedetomidine.

Although dosing information is unavailable in FAERS reports, other studies in the literature indicate that elevation of dexmedetomidine-associated bradycardia risk may be linked to dexmedetomidine overdose, either due to high loading doses, more concentrated infusions, or prolonged periods of sedation [19,26,27,28]. For instance, a multicenter, double-blind randomized controlled trial comparing the efficacy of dexmedetomidine with another sedative midazolam found that the incidence of bradycardia was approximately three times higher in the dexmedetomidine group compared to those sedated with midazolam (14.2% vs. 5.2%, *p* < 0.001) at a maintenance dose of 0.45 μg/kg/h [26]. Another double-blind, randomized controlled trial found that both the incidence (*p* = 0.027) and severity (*p* = 0.017) of bradycardia were significantly higher in those sedated with a 1.0 μg/kg loading dose compared to those administered a 0.8 μg/kg loading dose during rhinoplasty surgery [29]. Finally, a retrospective study found that a lower baseline heart rate (odds ratio = 0.89, 95% CI 0.82–0.96) and longer tourniquet time during surgery (odds ratio = 1.06, 95% CI 1.02–1.10) were both associated with an elevated likelihood of bradycardia development during spinal anesthesia [16]. Thus, patient status should be closely monitored for signs of bradycardia presentation, particularly for those requiring high initial loading dosages, longer periods of sedation, or those presenting with risk factors of bradycardia including lower initial heart rate and pre-existing cardiovascular complications.

Lactated Ringer’s solution is a mixture of sodium lactate, sodium chloride, potassium chloride, and calcium chloride dissolved in water that is used to stabilize blood volume and electrolyte imbalances in patients undergoing surgery [30,31]. Although generally considered safe with minimal contraindications and commonly administered to patients sedated with dexmedetomidine, Lactated Ringer’s solution may exacerbate bradycardia risk by promoting hyponatremia and reducing electrical conduction of the heart or by reducing blood pressure significantly and disrupting proper blood flow [30,32,33]. D-lactate has been shown to increase the activity of inducible nitric oxide synthase (iNOS) by promoting the transformation of macrophages to the M2 subtypes and elevating the expression of the vasodilator nitric oxide [34,35]. Likewise, dexmedetomidine has also demonstrated the ability to elevate nitric oxide levels by promoting the activation of endothelial nitric oxide synthase (eNOS); however, it is important to note that dexmedetomidine may also modulate the production of iNOS-derived nitric oxide [36,37]. Thus, concurrent administration of dexmedetomidine and Lactated Ringer’s solution may synergistically elevate bradycardia risk through overproduction of nitric oxide and prolonged vasodilation, particularly in those in which a large volume of Lactated Ringer’s solution is intravenously delivered or in patients with pre-existing cardiovascular conditions [30,34,36,37].

Bupivacaine is a local anesthetic used to modulate patient pain both during and after surgery [38,39]. Dexmedetomidine is commonly used as an adjuvant therapy to prolong postoperative pain reduction and shorten the time for sedation to occur [40,41,42]. However, multiple studies have demonstrated an increase in bradycardia risk when a combination therapy of dexmedetomidine and bupivacaine is used [42,43,44]. A randomized study of 100 American Society of Anesthesiologists patients found that the incidence of bradycardia in the group administered 3 mL of 0.5% bupivacaine followed by intravenous delivery of a 1 μg/kg loading dose of dexmedetomidine was significantly higher than the incidence of bradycardia in the control group only administered bupivacaine (33% vs. 4%, *p* < 0.001) [43]. Furthermore, a systematic review of 25 studies found that adjuvant administration of dexmedetomidine in those previously administered bupivacaine was associated with a 59% increase in relative risk of bradycardia (RR = 1.59, 95% CI 1.07–2.37) compared to those administered bupivacaine exclusively [42]. The increased production of nitric oxide by dexmedetomidine may potentiate the sedative effects of bupivacaine by increasing the risk of methemoglobinemia, a condition characterized by decreased oxygen transport capabilities that may lead to bradycardia [45,46]. Furthermore, higher doses of bupivacaine have been shown to promote stimulatory phosphorylation events of eNOS and elevation of nitric oxide levels [46].

Risperidone is an antipsychotic medication with indications for use in the treatment of schizophrenia and bipolar disorder [47]. Both dexmedetomidine and risperidone function as suppressants of the central nervous system by decreasing dopaminergic signaling in the case of dexmedetomidine or both dopaminergic and serotonin-mediated signaling in the case of risperidone [48,49,50,51,52]. Although the mechanism in which these two drugs interact to elevate bradycardia risk is not well characterized, it may be related to the additive effects on eNOS activity and overproduction of nitric oxide by macrophages [53].

Eight genes that regulate cardiac muscle contraction, including *COX5B*, *COX6A2*, *COX8B*, *MYH7*, *MYH6*, *MYL2*, *UQCRQ*, and *UQCR11*, were found to be downregulated in cardiac cells treated with dexmedetomidine. *COX5B*, *COX6A2*, and *COX8B* encode subunits of the cytochrome c oxidase complex in the mitochondrial electron transport chain [54,55]. Deficiencies in *COX6A2* have been linked to significant cardiac tissue remodeling and various cardiomyopathies including bradycardia [56,57]. *MYL2* encodes myosin light chain 2, a critical structural protein involved in the contraction of cardiovascular tissue with both missense mutations in this gene and downregulation of protein expression being associated with electrophysiological abnormalities, poor mechanical conduction, and bradycardia [58,59]. Likewise, downregulation of the sarcomeric structural proteins encoded by *MYH6* and *MYH7* have also been linked to impaired heart tissue contractility and bradycardia [60,61,62,63]. In vivo mechanistic studies have demonstrated significant bradycardia in *MYH6* knockout models while another study found that low expression levels of *MYH6* are associated with ischemic cardiomyopathies and heart failure [62,64]. In humans, *MYH7* may be the more dominant driver of the bradycardia phenotype, as it is highly expressed in the adult human heart, while *MYH6* is the predominant myosin isoform in mice [63,65]. Knockout of *URCRQ*, which encodes a prominent component of the mitochondrial electron transport chain, has shown to lead to the development of contractile dysfunction and reduced cardiomyocyte electrical activity due to disruptions in ATP production [66,67,68].

Proper cardiac health is dependent on the ability of the heart to rhythmically contract and relax to pump blood throughout the body and provide oxygen, among other nutrients, to systems throughout the body [69,70]. This process can be divided into multiple stages in which the electrical signals generated from the sinoatrial node promote the release of calcium from the sarcoplasmic reticulum, binding of calcium to the structural myosin proteins, sliding of myosin and actin filaments to drive cardiac contractility, and repolarization [69,70]. Because of the significant energetic requirements for the continual cycling of cardiac tissue between resting and contracting states, ATP production is closely linked to the ability of the heart to contract, with approximately 70% of all ATP produced by cardiocytes being directed toward this process [69,71]. Decreased ATP production and overall energetic deficiency may prolong the repolarization phase and extend the length of a single cardiac muscle contraction, reducing the overall number of full contractile cycles in a given period of time [71,72,73,74]. Thus, the suppression of mitochondrial genes related to energy production, such as *UQCRQ*, *UQCR11*, *COX5B*, *COX6A2*, and *COX8B*, may extend the length of a contractile cycle by prolonging the initiation of cardiac muscle contraction, thus lowering the heart rate and driving bradycardia progression. Similarly, suppression of structural genes, such as *MYH6*, *MYH7*, and *MYL2*, impairs the ability of myosin and actin filaments to associate and reduces the amount of contractile force generated, resulting in slower heart beats and bradycardia.

Proteomic analyses have also demonstrated impaired energetics and altered cardiac muscle contractility in multiple cardiovascular pathophysiologies. For instance, a study comparing the proteomic profiles of human hypertrophic cardiomyopathy found 88 differentially expressed proteins related to both contraction mechanisms, which included the downregulation of sarcomeric proteins MYH6 and MYBPC3, as well as the downregulation of metabolic proteins, including creatine kinase and the sarcoplasmic reticulum calcium transport protein ATP2A2 [75,76]. A proteomic analysis of sinoatrial node cells coupled with FAERS analysis of targets for bradycardia-associated drugs found seven downregulated proteins linked to contractile complications, including the potassium channels KCNH2, HCN1, and HCN4, as well as structural proteins such as CDH2 [77]. Finally, the upregulation of the vitamin-D-binding protein (VDB) in myocardial infarction samples may indirectly promote bradycardia by reducing cardiac concentrations of vitamin D, a crucial driver of calcium regulation and cardiac contractility, a deficiency of which has been linked to various arrhythmias including bradycardia [78,79,80]. Thus, there is a strong precedence of bradycardia phenotypes due to disruptions in cardiac energy production and contractility.

Despite these promising findings, the present study is not without limitations. First, due to the observational nature of the data derived from FAERS, a causal relationship between dexmedetomidine and an elevated risk of bradycardia cannot be determined. Consequently, the incidence of new bradycardia cases in those administered dexmedetomidine cannot be estimated. As many indirect reports were removed prior to the analysis (>1000), the ability of the study to identify safety signals for rare ADEs may be hindered, and the study may possibly be underpowered. However, as bradycardia was still the most frequently reported ADE among dexmedetomidine reports, the effects of this exclusion should be minimal with regard to estimating the strength of the association between dexmedetomidine and bradycardia risk. Furthermore, reports derived from the FAERS dashboard lack potentially important information such as dosage, which may influence whether dexmedetomidine is associated with an increase in the risk of bradycardia development, and they are subject to various biases including underreporting and other reporting biases. To combat this, future studies will utilize electronic health records to explore the role of potential confounders and refine risk estimates. In addition, potential DDIs identified in this study are hypothetical in nature and require additional clinical studies to validate their potential effects on bradycardia risk. Finally, the RNA-seq data utilized in this study were derived from mouse cardiac tissue, which may not be entirely comparable to human models due to physiological or genetic differences among species. In addition, the differentially expressed genes identified in this study may not be extrapolated across cell types or across species. Although these genes are functionally conserved between mice and humans, differences in kinetic requirements for proper cardiac function, mitochondria concentration, and the lack of more robust compensatory mechanisms in mice to modulate irregular heart rates may influence the risk of bradycardia between species treated with dexmedetomidine [81,82]. Nonetheless, this study further characterizes the safety profile of dexmedetomidine and provides potential mechanisms by which dexmedetomidine may elevate bradycardia risk in some patients. Further studies will seek to validate these potential DDIs and to determine differences in gene expression in human cells treated with dexmedetomidine to see whether different regulatory mechanisms modulate bradycardia risk in humans.

## 5. Conclusions

Bradycardia is a commonly reported adverse event in patients administered the sedative dexmedetomidine. Additional adverse events that co-occurred alongside bradycardia included syncope, loss of consciousness, cardiac arrest, and hypotension. Several potential DDIs were identified including Lactated Ringer’s solution, bupivacaine, and risperidone, which may elevate bradycardia risk through impaired cardiac contraction mechanisms and decreased mitochondrial energy production. Finally, eight downregulated genes mapped to cardiac muscle contraction were identified as possible regulators of dexmedetomidine-induced bradycardia, including *COX5B*, *COX6A2*, *COX8B*, *MYH7*, *MYH6*, *MYL2*, *UQCRQ*, and *UQCR11* in mouse cardiac cells.

## Figures and Tables

**Table 1:** A 2 × 2 contingency table for the validation of significant bradycardia-associated ADEs identified through association rule mining.

	With ADE 2	Without ADE 2
With Bradycardia	n_11_	n_12_
Without Bradycardia	n_21_	n_22_

**Table 2:** A 2 × 2 contingency table for the validation of significant DDIs identified through association rule mining.

	With Drug 2	Without Drug 2
With Bradycardia	n_11_	n_12_
Without Bradycardia	n_21_	n_22_

**Table 3:** Top 10 adverse drug events (ADEs) for the sedative dexmedetomidine.

Rank	Adverse Drug Event (ADE)	Frequency (n)
1	Bradycardia	199
2	Hypotension	124
3	Cardiac arrest	118
4	Off-label use	105
5	Agitation	67
6	Drug ineffective	58
7	Drug interaction	56
8	Product use issue	53
9	Oxygen saturation decreased	52
10	Pyrexia	48

**Table 4:** Top ADEs associated with bradycardia with lift ≥ 1.5 and count ≥ 10.

ADE	Support	Confidence	Coverage	Lift	Count
Syncope	0.0068	0.611	0.011	4.711	11
Loss of consciousness	0.0087	0.519	0.017	3.997	14
Cardiac arrest	0.0273	0.370	0.074	2.850	44
Hypotension	0.0286	0.359	0.079	2.770	46
Overdose	0.0062	0.303	0.020	2.336	10
Drug interaction	0.0106	0.279	0.038	2.148	17
Product administered to patient of inappropriate age	0.0068	0.234	0.029	1.804	11
Cardio-respiratory arrest	0.0062	0.233	0.027	1.793	10
Respiratory arrest	0.0093	0.203	0.046	1.562	15

**Table 5:** Disproportionality analysis of co-presenting ADEs identified by association rules.

ADE	Odds Ratio (OR)	Lower Bound of CI	Upper Bound of CI
Syncope	11.04	3.86	33.97
Loss of consciousness	7.65	3.28	17.98
Cardiac arrest	4.71	3.06	7.19
Hypotension	4.54	2.98	6.85
Overdose	3.01	1.26	6.69
Drug interaction	2.73	1.43	4.99
Product administered to patient of inappropriate age	2.11	0.95	4.32
Cardio-respiratory arrest	2.08	0.90	4.41
Respiratory arrest	1.76	0.91	3.22

**Table 6:** Top DDIs associated with bradycardia with lift ≥ 1.5 and count ≥ 10.

Drug	Support	Confidence	Coverage	Lift	Count
Lactated Ringer’s solution	0.0074	0.706	0.011	5.441	12
Bupivacaine	0.0074	0.387	0.019	2.984	12
Risperidone	0.0074	0.316	0.024	2.434	12
Albuterol	0.0081	0.250	0.032	1.927	13
Potassium chloride	0.0087	0.206	0.042	1.587	14
Haloperidol	0.0106	0.205	0.052	1.579	17
Sevoflurane	0.0130	0.196	0.066	1.513	21

**Table 7:** Disproportionality analysis of DDIs identified by association rules.

Drug	Odds Ratio (OR)	Lower Bound of CI	Upper Bound of CI
Lactated Ringer’s solution	16.96	5.49	62.19
Bupivacaine	4.43	1.93	9.78
Risperidone	3.22	1.46	6.74
Albuterol	2.32	1.11	4.53
Potassium chloride	1.79	0.90	3.35
Haloperidol	1.79	0.96	3.17
Sevoflurane	1.71	0.98	2.86

**Table 8:** Top 10 enriched pathways for downregulated genes in dexmedetomidine-treated mouse cardiac cells.

KEGG ^1^ Pathway	Num of Genes	Pathway Genes	Fold Enrichment	Enrichment FDR	Gene List
Ribosome	20	130	8.22	1.15 × 10^−10^	Rpl37, Rpl28, Rps15, Rps16, Rpl13a, Mrps12, Rpl8, Rpl13, Rpl7a, Mrps7, Rpl36, Rplp1, Rpl35, Rpl11, Rps10, Rplp2, Rpl38, Rpl41, Mrpl14, Rps9
Circadian rhythm	4	34	6.29	0.047	Cry1, Cry2, Dbp, Per1
Cardiac muscle contraction	8	87	4.92	0.0042	Cox5b, Cox6a2, Cox8b, Myh7, Myh6, Myl2, Uqcrq, Uqcr11
Oxidative phosphorylation	12	133	4.82	0.00029	Cox5b, Cox6a2, Cox8b, Ndufa2, Uqcrq, Atp5g3, Atp5d, Ndufa12, Uqcr11, Atp5e, Atp5g2, Ndufv3
Mitophagy-animal	6	68	4.72	0.026	Cited2, Rras, Src, Ubb, Map1lc3a, Pink1
Acute myeloid leukemia	6	70	4.58	0.027	Cd14, Cebpa, Eif4ebp1, Myc, Pim1, Tcf7l1
Parkinson’s disease	21	262	4.28	3.40 × 10^−6^	Slc25a4, Cox5b, Cox6a2, Cox8b, Ube2j2, Itpr3, Klc2, Ndufa2, Dusp1, Sod1, Ubb, Uqcrq, Atp5g3, Park7, Atp5d, Ndufa12, Uqcr11, Atp5e, Atp5g2, Pink1, Ndufv3
Chemical carcinogenesis-ROS	17	220	4.13	6.08 × 10^−5^	Slc25a4, Cox5b, Cox6a2, Cox8b, Ephx1, Ndufa2, Sod1, Src, Uqcrq, Atp5g3, Atp5d, Ndufa12, Mgst3, Uqcr11, Atp5e, Atp5g2, Ndufv3
Hypertrophic cardiomyopathy	7	91	4.11	0.026	Des, Myh7, Itga3, Lmna, Myh6, Myl2, Tgfb1
Coronavirus disease-COVID-19	18	235	4.09	4.42 × 10^−5^	Rpl37, C1qa, Rpl28, Rps15, Rps16, Rpl13a, Rpl8, Rpl13, Rpl7a, Rpl36, Rplp1, Rpl35, Rpl11, Rps10, Rplp2, Rpl38, Rpl41, Rps9

^1^ KEGG: Kyoto Encyclopedia of Genes and Genomes.

## Data Availability

The original contributions presented in this study are included in the article. Further inquiries can be directed to the corresponding author.

## Abbreviations

The following abbreviations are used in this manuscript:

NCBI	National Center for Biotechnology Information
GEO	Gene Expression Omnibus
DDI	Drug–drug interaction
OR	Odds ratio
CI	Confidence interval
FDR	False discovery rate
FDA	Food and Drug Administration
FAERS	FDA Adverse Event Reporting System
ADE	Adverse drug event
BPM	Beats per minute
ATP	Adenosine triphosphate
MYBPC3	Cardiac myosin-binding protein C
ATP2A2	Sarcoplasmic reticulum calcium ATPase 2
KCNH2	Potassium voltage-gated channel subfamily H member 2
HCN1	Hyperpolarization-activated cyclic nucleotide-gated potassium channel 1
HCN4	Hyperpolarization-activated cyclic nucleotide-gated potassium channel 4
CDH2	N-cadherin
VDB	Vitamin-D-binding protein
iNOS	Inducible nitric oxide synthase
eNOS	Endothelial nitric oxide synthase
KEGG	Kyoto Encyclopedia of Genes and Genomes
COX5B	Cytochrome c oxidase subunit 5B
COX6A2	Cytochrome c oxidase subunit 6A2
COX8B	Cytochrome c oxidase subunit 8B
MYH6	Myosin heavy chain, α isoform
MYH7	Myosin heavy chain, β isoform
MYL2	Myosin regulatory light chain 2
UQCRQ	Ubiquinol-cytochrome c reductase, complex III subunit VII
UQCR11	Ubiquinol-cytochrome c reductase, complex III subunit XI
